# New Class of Crosslinker-Free Nanofiber Biomaterials from *Hydra* Nematocyst Proteins

**DOI:** 10.1038/s41598-019-55655-0

**Published:** 2019-12-13

**Authors:** Theresa Bentele, Federico Amadei, Esther Kimmle, Mariam Veschgini, Philipp Linke, Mariana Sontag-González, Jutta Tennigkeit, Anthony D. Ho, Suat Özbek, Motomu Tanaka

**Affiliations:** 1 Centre for Organismal Studies, Department of Molecular Evolution and Genomics, Heidelberg University, 69120 Heidelberg, Germany; 2 Physical Chemistry of Biosystems, Institute of Physical Chemistry, Heidelberg University, 69120 Heidelberg, Germany; 3 Department of Medicine V, University of Heidelberg, 69120 Heidelberg, Germany; 4 Center for Integrative Medicine and Physics, Institute for Advanced Study, Kyoto University, 606-8501 Kyoto, Japan; 5 Present Address: School of Earth and Environmental Sciences, Science Medicine and Health, University of Wollongong, NSW 2522 Wollongong, Australia

**Keywords:** Biomaterials, Soft materials

## Abstract

Nematocysts, the stinging organelles of cnidarians, have remarkable mechanical properties. *Hydra* nematocyst capsules undergo volume changes of 50% during their explosive exocytosis and withstand osmotic pressures of beyond 100 bar. Recently, two novel protein components building up the nematocyst capsule wall in *Hydra* were identified. The cnidarian proline-rich protein 1 (CPP-1) characterized by a “rigid” polyproline motif and the elastic Cnidoin possessing a silk-like domain were shown to be part of the capsule structure via short cysteine-rich domains that spontaneously crosslink the proteins via disulfide bonds. In this study, recombinant Cnidoin and CPP-1 are expressed in *E. coli* and the elastic modulus of spontaneously crosslinked bulk proteins is compared with that of isolated nematocysts. For the fabrication of uniform protein nanofibers by electrospinning, the preparative conditions are systematically optimized. Both fibers remain stable even after rigorous washing and immersion into bulk water owing to the simultaneous crosslinking of cysteine-rich domains. This makes our nanofibers clearly different from other protein nanofibers that are not stable without chemical crosslinkers. Following the quantitative assessment of mechanical properties, the potential of Cnidoin and CPP-1 nanofibers is examined towards the maintenance of human mesenchymal stem cells.

## Introduction

**Figure 1:**
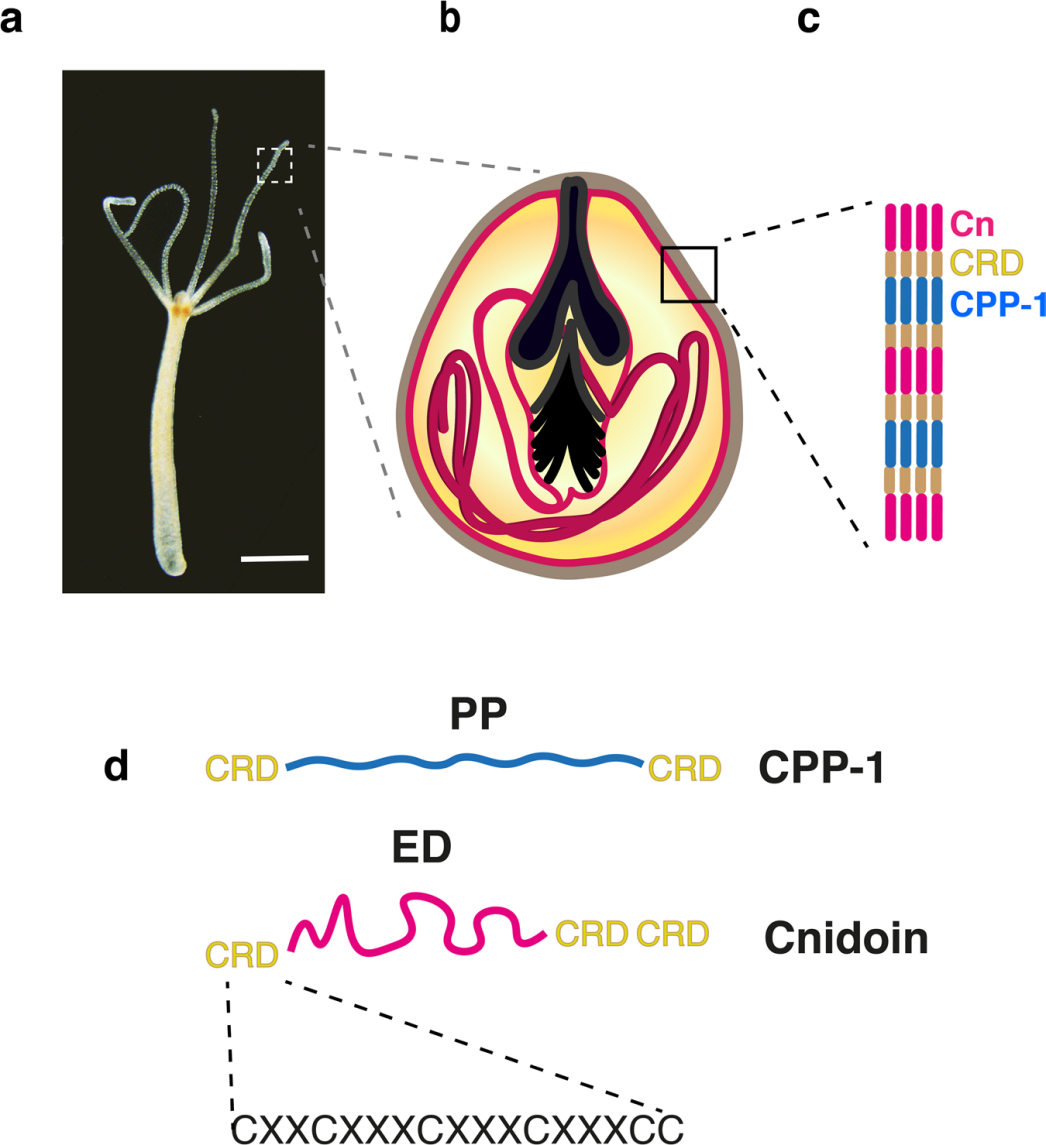
(**a**) Bright field image of a *Hydra* polyp (scale bar: 500 µm). (**b**) Schematic representation of a stenothele-type nematocyst with a large stylet apparatus and a coiled tubule inside of the hollow capsule body. (**c**) The nematocyst capsule wall consists of CPP-1 and Cnidoin (Cn), linked via cysteine-rich domains (CRDs). (**d**) CPP-1 has a “rigid” polyproline domain (PP) flanked by two CRD units, while Cnidoin consists of an “elastic”, silk-like domain (ED) flanked by CRD units. Each CRD unit has six cysteine residues in a conserved pattern (X denotes a non-cysteine residue).

Nematocysts are harpoon-like organelles characteristic of the cnidarian phylum^1^. The development of *Hydra* nematocysts, which comprise four different types, occurs in the body column of the polyps in specialized cells, called nematocytes. After maturation, nematocytes migrate towards the tentacles and are mounted in so called “battery cells” (Fig. 1a)^2^. Nematocysts consist of a hollow capsule body, to which an inverted tubule is attached that in the case of the large “stenothele” type of nematocyst has a stylet used to perforate the prey’s integument and allow injection of peptide toxins to paralyze the prey (Fig. 1b)^3–6^.

As biomaterials, one of the unique characteristics of nematocysts is the outstanding mechanical toughness of the capsule wall structure. Maturation of the capsule involves “wall hardening” and build-up of an internal osmotic pressure of about 150 bar. After discharge, the elastically stretched nematocyst capsule shrinks to 50% of its original volume signifying the release of kinetic energy during the explosive exocytosis^7^. Actually, the nematocyst discharge is one of the fastest events in the animal kingdom, generating an acceleration of more than 5 million g^8,9^. The nematocyst capsule comprises protein complexes crosslinked by intermolecular disulfide bonds between cysteine-rich domains (CRDs), which are found at both C- and N-termini of various nematocyst proteins (Fig. 1c)^10^. Among those, minicollagens are major structural proteins possessing short collagen sequences (Gly-X-Y) flanked by polyproline stretches and terminal CRDs^11^. Previous data on nematocyst proteins containing CRDs have demonstrated that these are tightly integrated due to disulfide reshuffling into the capsule polymer and can only be released as monomers by reducing agents^7,12–15^. We have recently demonstrated that the CRD can be used as a versatile crosslinker module to create linear or branched polymers from diverse proteins^10^.

In our proteome study of *Hydra* nematocysts, two new capsule proteins flanked by terminal CRDs have been identified; cnidarian proline-rich protein 1 (CPP-1) and Cnidoin (Fig. 1d)^7,16^. CPP-1 has a continuous polyproline (PP) stretch forming a rigid polyproline II helix like the collagen sequence, but is not able to induce a triple helix. Cnidoin possesses an elastic, silk-like sequence instead of a rigid collagen-like PP motif (Fig. 1d)^7^. The combination of “rigid” CPP-1 and “elastic” Cnidoin therefore seems to be a very promising strategy for the design of new biomaterials that are capable of forming stable structures via spontaneous crosslinking and realize outstanding toughness and flexibility as nematocyst capsules have.

Protein fibers in nature, such as silks of spiders and silkworms, were produced by the enforced passage of concentrated protein solutions through spinnerets, resulting in fibers with diameters of some µm to some tens of µm^17^. For example, the dragline silk of spiders has been drawing increasing attention as a unique biomaterial possessing a supercontracting capability in contact with water^18,19^. Electrospinning is a commonly used method to fabricate thin fibers based on silk proteins^20,21^, collagen^22,23^, and gelatin^24^ for various applications, including wound healing and tissue engineering^25–28^. Although the natural dragline spider silks do not need additional chemical crosslinks, commonly used protein and polymer fibers require covalent crosslinks for the material applications in water. However, it has been reported that even a trace amount of commonly used chemical crosslinkers, such as glutaraldehyde, showed cytotoxicity^29^. Alternative strategies include the coupling of photocrosslinkable side chains^30^ or mixing of synthetic polymers^31,32^. Woolfson *et al*. proposed the crosslinker-free synthesis of complementary pairs of peptides that spontaneously self-assembled into highly ordered nanofibers by the combination of elastrostatic interactions, hydrogen bonds, and hydrophobic interactions^33^.

As biomaterials, protein nanofibers have also been drawing increasing attentions as an artficial matrix for culturing stem cells. Liu *et al*. have demonstrated that very thin (270 nm), chemically crosslinked gelatin nanofiber substrates can be used for the long-term culture of human pluripotent stem cells^34^. More recently, they have shown that nanofiber substrates are advantageous over commonly used matrigel cultures to discriminate populations of different pluripotent stem cells, which can be attributed to the fact that the adhesion of stem cells to nanofibers is much weaker compared to the adhesion onto gel substrates^35^.

In this study, we introduce the synthesis of a new class of crosslinker-free nanofibers based on *Hydra* nematocyst proteins Cnidoin and CPP-1 by electrospinning. Owing to the spontaneous crosslinking capability of CRDs, we systematically optimized the preparative conditions and fabricated crosslinker-free protein nanofibers that are stable under water, which can potentially be used for the culture of human stem cells.

## Materials

Unless stated otherwise, solvents and chemicals were purchased from Sigma-Aldrich (Munich, Germany) and used without further purification. Double deionized water (MilliQ, Merck, Darmstadt, Germany) with a specific resistivity > 18 MΩ∙cm was used for the buffer preparation. In this study, the following buffer solutions were used: (i) PBS buffer; 136.9 mM NaCl, 2.7 mM KCl, 8.9 mM Na_2_HPO_4_, 1.8 mM KH_2_PO_4_, (ii) PBST; 0.1 wt% Tween20 (Roth, Karlsruhe, Germany) in PBS. *Hydra* medium; 1.0 mM Tris HCl (pH 7.6) (Roth, Karlsruhe, Germany), 1.0 mM NaHCO_3_, 0.1 mM KCl, 0.1 mM MgCl_2_ (AppliChem, Darmstadt, Germany), 1.0 mM CaCl_2_ (Roth, Karlsruhe, Germany) in Elix-H_2_O. *Hydra* nematocyst isolation buffer; 50% (v/v) Percoll (GE Healthcare, Uppsala, Sweden), 10 wt% sucrose (J.T. Baker, Deventer, Holland), 0.003 wt% Triton X-100 in MilliQ H_2_O). Spinning solution; 250 mM imidazole, 0.4 M L-arginine, 10 mM DTT in PBS, freshly prepared before use.

## Methods

### Animal culture, nematocyst isolation and immunocytochemistry

*Hydra magnipapillata*^36^ were cultured in *Hydra* medium at 18 °C and fed twice a week with freshly hatched *Artemia salina* nauplii. Animals used for the experiments were starved for 24 h. Intact nematocysts were isolated from whole *Hydra* animals following the protocol reported previously^37^. In brief: animals kept at −80 °C overnight were thawn and suspended in nematocyst isolation buffer, followed by centrifugation at 4 °C and 7500  g for 15 min (2×). Then the pellet was resuspended in 10% sucrose (w/v) and 0.003% Triton X-100 (w/v) in PBS and centrifuged for 10 min at room temperature. For immunocytochemistry animals were relaxed in 2% urethane  and then fixed in Lavdovsky’s fixative (50% ethanol, 10% formaldehyde, 4% acetic acid, 36% water by volume) overnight at 4 °C. The fixative was removed by washing the samples three times with PBS for 10 min. A second fixation was performed using 4% paraformaldehyde (w/v) in PBS for 30 min at RT. The fixative was removed and the animals were washed 3× for 10 min using 0.5% Triton X-100 in PBS. After a 30 min blocking step with 0.5% bovine serum albumin, BSA (Roth, Karlsruhe, Germany) (w/v) in PBST at RT, the animals were incubated overnight at 4 °C with anti-Cnidoin (guinea pig, 1:250)^7^ and polyclonal anti-CPP-1 (rat, 1:250) antibodies (Eurogentec, Lüttich, Belgium) diluted in blocking solution. The CPP-1 antibody was raised against a peptide comprising the N-terminal CRD domain (CPAPCGGDLNCWPTCDATCC). After washing 3× in PBST, the animals were incubated with donkey anti-rat Alexa Fluor 488 (Thermo Fisher, Waltham, MA, USA) (1:1000) or goat anti-guinea pig IgH (H + L) Alexa Fluor 647 (Invitrogen, Waltham, MA, USA) (1:1000) for 2 h at RT. Cell nuclei were stained with 4′,6-diamidino-2-phenylindole, DAPI (Roche, Basel, Switzerland) (1:1000) in PBST for 10 min at RT. Animals were washed 3× with PBST and mounted on coverslips with 90% glycerol (w/v) in PBS. Confocal fluorescence microscopy imaging was performed on a Nikon ECLIPSE Ti (Nikon, Düsseldorf, Germany), and images were analyzed using the NIS Elements software.

### Expression of Cnidoin and CPP-1 in bacteria, Western blot

The recombinant expression of Cnidoin and CPP-1 in *E. coli* BL21 (DE3) was performed from a pET21b vector, which introduces a C-terminal polyhistidine tag. The amino acid sequences of reCPP-1 and reCnidoin are presented in Supporting Information S1. After lysis, both reCPP-1 and reCnidoin were purified from cell pellets under denaturing conditions (8 M urea) using Ni-NTA beads. For reCnidoin, extensive washing cycles were performed as it was exclusively found in inclusion bodies, whereas reCPP-1 was partly soluble. Isolated *Hydra* nematocyst samples were denatured by heating (95 °C, 10 min) with or without 2-mercaptoethanol. The lysate was prepared by dissolving one polyp in reducing or non-reducing sample buffer by heating and vortexing. Samples were separated by sodium dodecyl sulfate polyacrylamide gel electrophoresis (SDS-PAGE) using 12% gels and transferred to nitrocellulose membranes by wet blotting. Unspecific binding was blocked by incubating the membrane with 5% milk powder (w/v) in PBST. After 3 × 10 min washing cycles with PBST, the nitrocellulose membrane was incubated with anti-Cnidoin and anti-CPP-1 (1:1000) antibodies overnight at 4 °C, followed by 3 × 10 min washing steps. The primary antibodies were detected by incubation with secondary antibodies coupled to horseradish peroxidase (HRP) for 1 h and blots were developed using a peroxidase substrate for enhanced chemiluminescence.

### Fabrication of reCnidoin and reCPP-1 nanofibers by electrospinning

The crucial parameters for the fabrication of protein nanofibers are to achieve (i) a high protein concentration and (ii) a high viscosity. To increase the protein concentration for reCPP-1 and reCnidoin, which tended to form agglomerates by spontaneous CRD oxidation, precipitated protein pellets were dissolved in a minimal volume of 200 mM DTT solution. This enabled us to achieve a protein concentration of about 40 mg/mL as estimated by photometric analysis at 280 nm. To increase the viscosity of the protein solution,we added a high molecular weight polyethylene glycol (PEG, Mw = 900 kDa) to the final concentration of 4% (w/v), at which the formation of a continuous PEG nanofiber was reported^38^. The fibers were produced by means of electrospinning^39,40^, using a NANON-3 (MECC, Fukuoka, Japan). The solution was pumped at a speed of 20 µL/min through a stainless-steel blunt needle (∅ ≈ 27 G) pointing vertically down to an aluminum foil collector plate. The bias voltage of 17 kV was applied over 11 cm distance vs. the electrically grounded collector plate. Throughout the experiments the relative humidity was regulated at less than 30% by the spinning of fibers in a sealed chamber filled with nitrogen gas.

### Atomic force microscopy (AFM)

AFM measurements were performed by using a NanoWizard 3 AFM (JPK, Berlin, Germany). Antimony doped silicon quadratic pyramidal tips (RTESPA-150, Bruker, USA) and silicon nitride quadratic pyramidal tips (MLCT, Bruker, USA) whose vertical spring constants have a nominal value of 6 N/m and 0.03 N/m were used in contact mode in air and in PBS, respectively. The spring constant of a cantilever was determined prior to the experiments using the thermal noise method. In case of *Hydra* nematocysts, a small portion of nematocyst suspension was shortly dried in ambient atmosphere, and rehydrated in PBS. Purified proteins were covalently anchored on glass slides pre-coated with 3-glycidoxypropyltrimethoxysilane^41^. Here, the protein solution was spread on the silanized slides and incubated over night at 4 °C. In the case of protein nanofibers, the fibers deposited on glass substrates were measured (i) in air, (ii) in air after washing, and (iii) in buffer. The surface topography was scanned on a region 50 × 50 µm^2^ at a line rate of 0.1 Hz. The mechanical properties of capsule walls, purified proteins, and nanofibers were characterized by AFM nanoindentation of 9 × 9 µm^2^ region. The measured force-distance curves were analyzed using the Bilodeau model for pyramidal indenters^42,43^. The effective elastic moduli presented in the following were obtained from *N* > 100 force-indentation curves.

### Human mesenchymal stem cells (hMSC)

hMSCs were isolated and cultured as described before^44^. Briefly, bone marrow from healthy donors for allogeneic transplantation was taken after written consent using guidelines approved by the Ethic Committee on the Use of Human Subjects at the University of Heidelberg. The cells were cultured in MSCGM (Mesenchymal Stem Cell Growth Medium, Lonza, Basel, Switzerland) at 37 °C in a humidified atmosphere and the culture medium was exchanged every second day. Protein nanofibers were prepared as described above, rinsed with 70% ethanol (v/v) two times and dried. Then hMSC were seeded onto the nanofibers at a density of 500 cells/cm^2^ and fixed with 2% paraformaldehyde (w/v) after 20 days. For immunostaining, the cells were incubated with mouse anti-STRO-1 antibody followed by goat anti-mouse antibody conjugated with Alexa Fluor 488 for 1 h each (Thermo Fisher, Waltham, MA, USA), while cell nuclei were stained with DAPI. Images were acquired with a Zeiss Axio Observer equipped with a 20× air objective with phase contrast.

## Results

### CPP-1 and Cnidoin are structural proteins of the *Hydra* nematocyst wall

**Figure 2:**
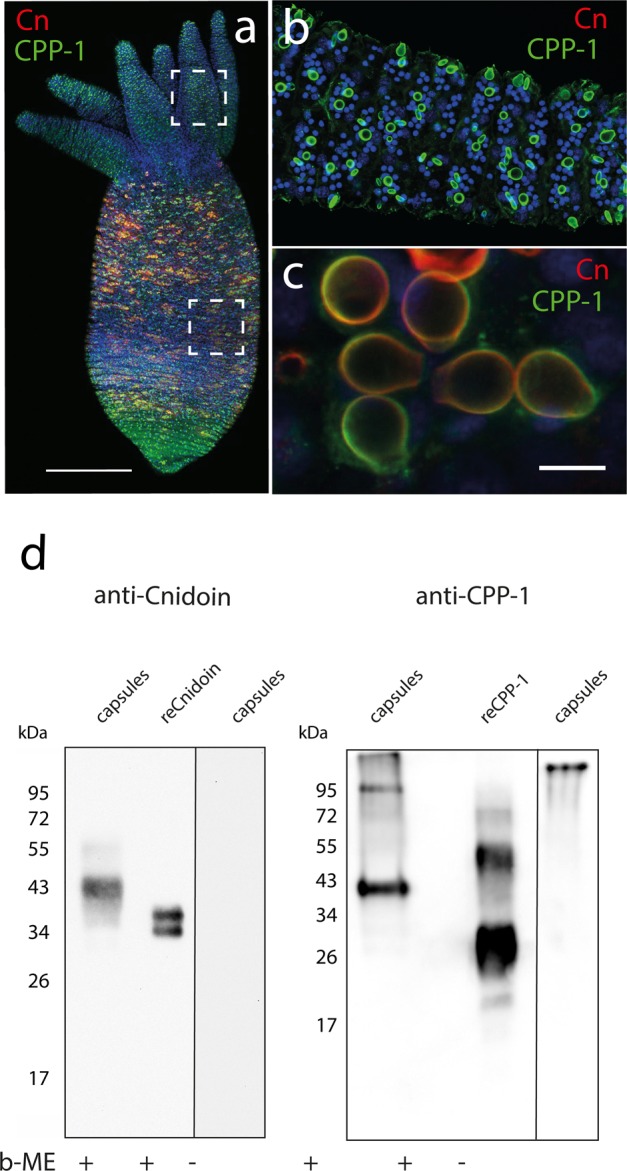
(**a**) Immunofluorescence image of a *Hydra* polyp stained with CPP-1 and Cnidoin antibodies; cell nuclei (blue), CPP-1 (green), and Cnidoin (red). (**b**) Mature capsules in tentacles showed only CPP-1 signals. (**c**) Zoom-in images of capsules in the gastric region indicated co-localization of CPP-1 and Cnidoin in nematocyst walls. (**d**) Western blot analysis of CPP-1 and Cnidoin in isolated nematocysts and after recombinant expression in *E. coli* (reCPP-1, reCnidoin). (+) and (−) indicate the presence or absence of β−mercaptoethanol (β−ME) in the sample buffer. Uncropped images of gels are presented in Figure S4.

Figure 2a represents an immunofluorescence image of a *Hydra* whole mount with CPP-1 (green) and Cnidoin (red) antibodies. The overlapping signals can be detected in the body column region, corresponding to developing nematocyte nests. A zoom-in image of this region (Fig. 2c) clearly indicated the co-localization of Cnidoin and CPP-1 in the capsule wall. Intriguingly, signals for CPP-1 could be detected also in the tentacle region, which contains only mature nematocysts whose dense protein polymer normally prevents antibody detection (Fig. 2b). This suggests that Cnidoin is more densely packed in mature nematocyst walls compared to CPP-1 and hence the accessibility of antibodies is spatially restricted. In fact, our recent studies on minicollagen CRDs suggested that the C-terminal CRD fold (C-CRD) is able to participate in several disulfide bonds, whereas the N-terminal CRD fold (N-CRD) is strictly monovalent^10^. Therefore, it is plausible that CPP-1 possessing N-CRD type CRDs at both termini is less packed compared to Cnidoin that has C-CRD type folds in both C-terminal domains^7^.

Figure 2d represents a Western blot of isolated nematocyst capsules and recombinant proteins produced in *E. coli*. The details of the PCR protocols and the gel images of PCR products are presented in Supporting Information S3. The samples were loaded with ( + ) or without (−) β-mercaptoethanol (β-ME) for the reductive cleavage of disulfide bonds. The CPP-1 protein detected in isolated capsules migrated with a significantly higher molecular weight (≈ 42 kDa) (Fig. 2d) than theoretically predicted for the mature protein (26 kDa), signifying considerable posttranslational modifications of CPP-1 in *Hydra* (Supporting Information S2). We assume that apart from N- and O-glycosylation, proline hydroxylation adds to this shift by generally decreasing electrophorectic mobility of CPP-1 as described before for other proteins^45^. This was confirmed by the detection of CPP-1 protein expressed in *E. coli* (hence termed reCPP-1), which showed an apparent molecular weight of about 27 kDa (Fig. 2d). The Cnidoin band detected in isolated capsules migrated at about 42 kDa in accordance with our previous data^7^. As already reported by Beckmann *et al*. recombinant Cnidoin (hence termed reCnidoin) showed a double band at 38 kDa, suggesting partial reoxidation of the CRDs and conformational rearrangement during SDS-PAGE^7^. CPP-1 and Cnidoin proteins in isolated capsules could not be detected in the absence (−) of reducing agents, since the highly crosslinked protein oligomers were not able to enter the polyacrylamide gel (Fig. 2d). These results confirmed that both CPP-1 and Cnidoin are structural proteins of the nematocyst wall, integrated during morphogenesis by disulfide crosslinking of CRDs.

### Mechanical properties of *Hydra* nematocysts and bulk proteins

**Figure 3:**
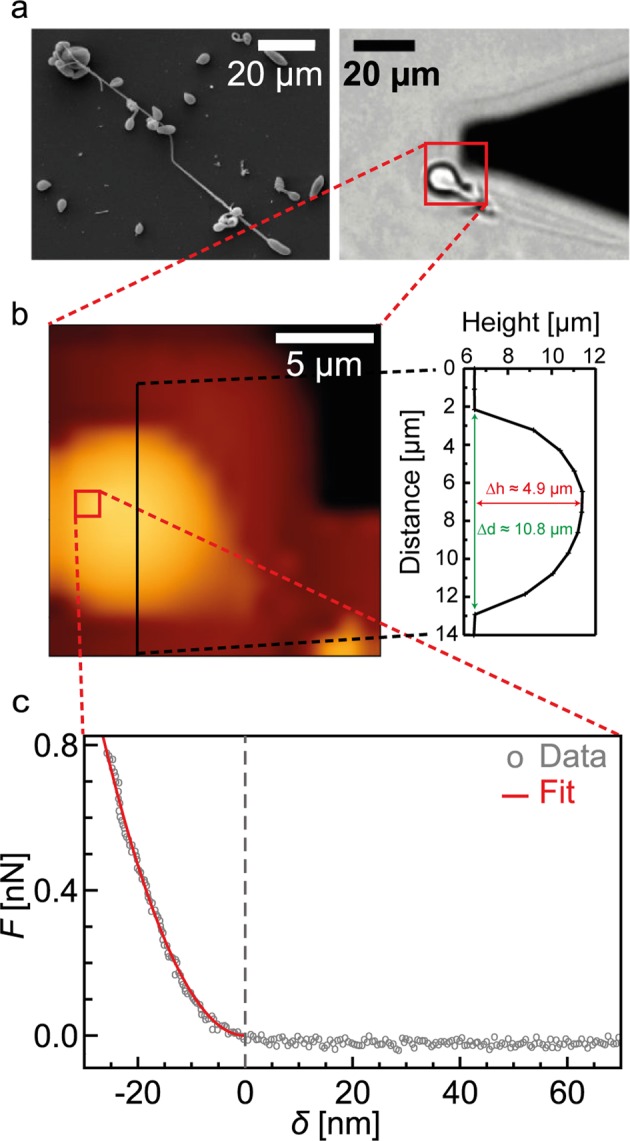
(**a**) Left: SEM image of isolated, partly discharged nematocysts. Right: Bright field microscopy image of an isolated discharged stenothele. The black triangle shadow corresponds to the AFM cantilever. (**b**) Height map of the discharged nematocyst collected from the red square in (**a**) (17 × 17 µm^2^). (**c**) A typical force-indentation curve measured on the nematocyst at the position indicated by the red square in (**b**) (1.1 × 1.1 µm^2^). The force-indentation data (gray circles) was fitted with the Bilodeau model for pyramidal tips (red curve).

The left panel of Fig. 3a shows a scanning electron microscopy (SEM) image of isolated *Hydra* nematocysts, partly showing discharged tubules. The right panel represents the bright field image of a discharged stenothele-type nematocyst adherent on the surface of a glass substrate for AFM measurement. Undischarged nematocysts were not used for the measurements as they retain the osmotic pressure after isolation. The triangular “shadow” is the AFM cantilever. Figure 3b represents the surface topography of the nematocyst, which is indicated by a red square in Fig. 3a (17 × 17 µm^2^). A typical force-indentation curve from the region highlighted in Fig. 3b (1.1 × 1.1 µm^2^) measured in physiological PBS and the corresponding fit with the Bilodeau model are shown in Fig. 3c. As presented in Supporting Information S5, the distribution of elastic moduli extracted from *N* > 100 curves is well fitted with a log-normal function. The mean and the standard deviation of effective elastic modulus of the capsule was *E*_capsule_ = 2.0 ± 2.4 MPa. Note that the validation of the obtained elastic modulus is not easy, because there has been no report on the mechanical properties of nematocyst capsules. Thus, to gain further insights on the mechanics of nematocyst capsule proteins, we measured the elasticity of purified recombinant CPP-1 and Cnidoin expressed in *E. Coli*. Once the solution of purified reCPP-1 or reCnidoin was deposited on the glass substrate coated with glycidoxypropyltrimethoxysilane, the proteins formed bulk solids by spontaneous crosslinking of CRDs. The AFM indentation in physiological PBS yielded the bulk elastic modulus of reCPP-1 and reCnidoin blocks, *E*_CPP-1(R)_ = 7.8 ± 9.0 MPa and *E*_Cnidoin(R)_ = 2.3 ± 2.0 MPa, respectively. The distribution of each protein extracted from *N* > 100 force-indentation curves followed log-normal distributions, confirming the statistical reliability of the data (Supporting Information S6). The obtained result suggested that the bulk reCnidoin protein is softer than the bulk reCPP-1, which can be attributed to its disordered silk-like motif.

### Optimization of nanofiber production

Since the viscosity of the pure protein solutions was *η*_noPEG_ ≈ 2 × 10^−3^ Nm^−2^s, we added PEG 900 kDa for higher viscosity. The viscosity of PEG solution was measured as a function of the concentration of PEG 900 kDa by a self-built falling ball viscometer (Supporting Information S7). At [PEG] = 4% (w/v), the viscosity of PEG solution increased by about three orders of magnitude to *η*_4%PEG_ ≈ 2 × Nm^−2^s, which is in good agreement with a previous report^46^. We observed that the spinning of the 2% (w/v) solution resulted in discontinuous beads, while the 4% (w/v) solution formed continuous nanofibers (Supporting Information S7). Moreover, [PEG] = 4% (w/v) lies between the overlap concentration of 1 MDa PEG (c* ≈ 1.6% (w/v)) and the critical transition concentration from a semi-diluted solution to a concentrated solution (c* ≈ 4.9% (w/v))^46^. As shown in Supporting Information S8, we also systematically investigated the influence of ionic strength and relative humidity (r.h.), and found that the relative humidity significantly influenced the quality of nanofibers. Continuous, homogeneous nanofiber formation could be observed in dry atmosphere (r.h. < 25%), while the electrospinning in humid atmosphere (r.h. = 85% and 50%) resulted in discontinuous beads^47^. In contrast, the ionic strength and hence the conductivity of spinning solutions showed almost no influence. Therefore, in the following, we fabricated all the nanofibers at [PEG] = 4 wt% and r.h. < 25%.

### Structures and mechanical properties of reCPP-1 and reCnidoin nanofibers

**Figure 4:**
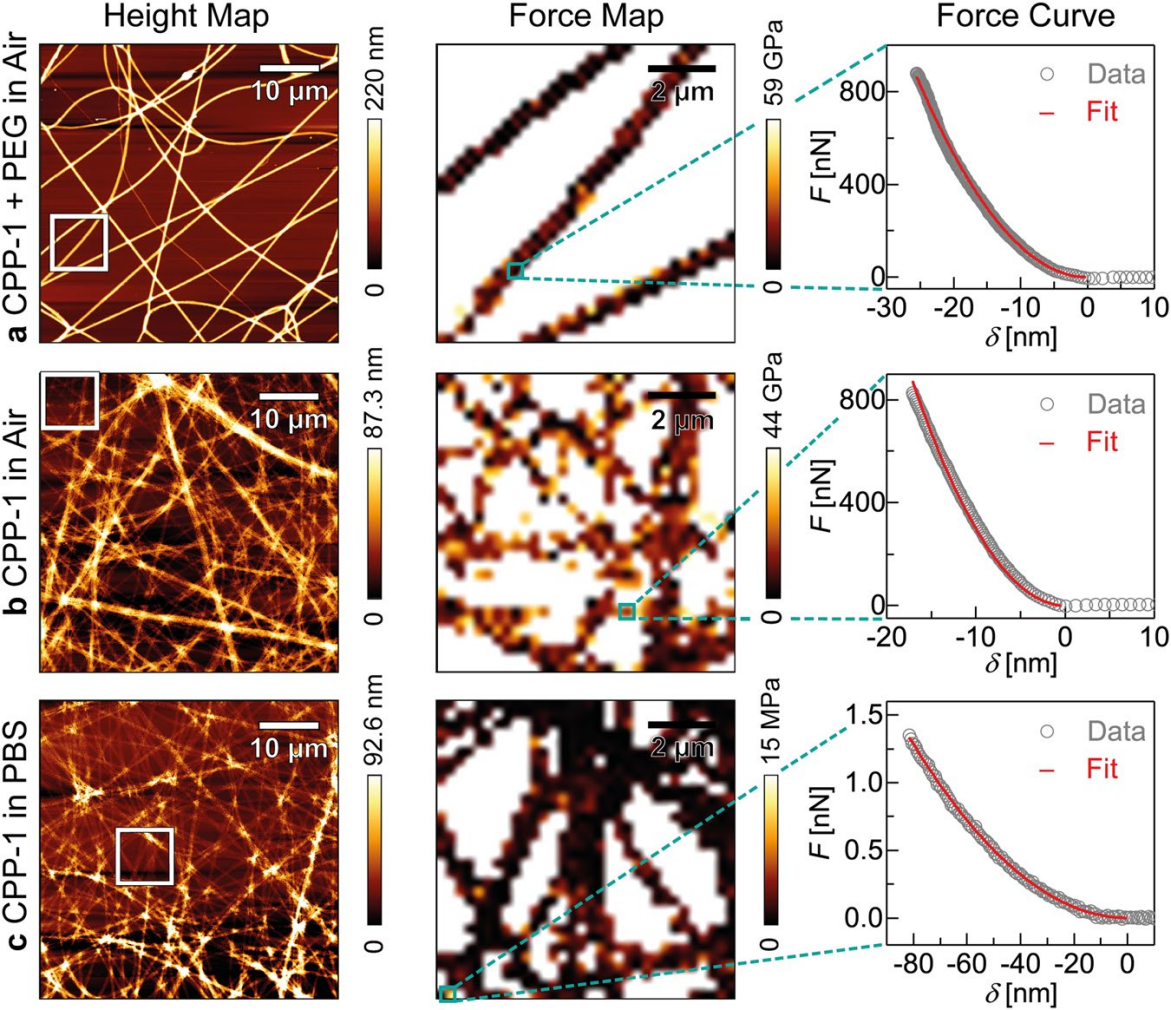
AFM measurements of electrospun reCPP-1 fibers. First, a reCPP-1:PEG (1:1) mixture was electrospun and characterized in air (**a**). Second, the reCPP-1:PEG fibers were washed by water, and the remaining reCPP-1 fibers were characterized in air (**b**), as well as in PBS (**c**). Each dataset consists of height maps (left column), force maps (middle column), and characteristic force-indentation curves (right column) fitted with the Bilodeau model (red curve).

Based on the above-mentioned results, we fabricated protein nanofibers by electrospinning the protein-PEG solution on glass coverslips. Figure 4 represents the maps of surface topography (left column), elastic modulus (central column), and a characteristic force-indentation curve (right column) of reCPP-1 nanofibers (a) in air, (b) in air after washing with water, and (c) in PBS. As shown in the left panel of Fig. 4a and Supporting Information S9, the freshly spun reCPP-1-PEG nanofibers showed a uniform width (*w* ≈ 810 ± 164 nm) and height (*h* ≈ 148 ± 16 nm) over a 50 × 50 µm^2^ area. The map of apparent elastic moduli collected from a 9 × 9 µm^2^ region (Fig. 4a, center) also exhibited a uniform elastic modulus, and the force-indentation curve, exemplified in the right panel, can be well fitted with the Bilodeau model (red curve). The mean value and the standard deviation of the apparent elastic moduli obtained from *N* > 100 indentation measurements was *E*_CPP-1 + PEG, air_ = 4.1 ± 3.5 GPa. After washing the sample with water and drying it in ambient atmosphere, the same sample was subjected to the indentation measurements in air (Fig. 4b and Supporting Information S9). We observed a distinct thinning of individual fibers, *w* ≈ 521 ± 173 nm and *h* ≈ 7 ± 4 nm, respectively. This suggests the removal of non-crosslinked PEG from the nanofibers, since PEG has no functional group to chemically bind to reCPP-1 or reCnidoin. The effective elastic modulus of reCPP-1 fibers after the removal of water, *E*_CPP-1, air_ = 6.6 ± 8.3 GPa, is 1.6 times larger than that of reCPP-1-PEG nanofibers, which seems reasonable from the elastic modulus of pure PEG fibers, *E*_PEG, air_ = 1.6 ± 1.8 GPa (Supporting Information S10). The topographic profile in PBS (Fig. 4c and Supporting Information S9) implied that the thickness of reCPP-1 nanofibers was *h* ≈ 7 ± 3 nm. Remarkably, the elastic modulus of reCPP-1 nanofibers in physiological PBS, *E*_CPP-1, PBS_ = 0.7 ± 1.2 MPa, was almost four orders of magnitude smaller than the one in air, *E*_CPP-1, air_ = 6.6 ± 8.3 GPa. Actually, this value is even one order of magnitude smaller than the corresponding value of bulk reCPP-1 protein in physiological PBS, *E*_CPP-1(R)_ = 7.8 ± 9.0 MPa, which suggests that reCPP-1 in nanofibers after the removal of PEG is less packed compared to the bulk reCPP-1. As presented in Supporting Information S11, the distribution of each elastic modulus out of *N* > 100 indentation results can be well fitted with log normal function, confirming the statistical reliability.

Along the same line, the surface topography, the elasticity map, and a characteristic force-indentation curve of reCnidoin nanofibers were measured (a) in air, (b) in air after washing with water, and (c) in physiological PBS (Supporting Information S12, S13, S14). The width and thickness of reCnidoin-PEG nanofibers seemed comparable to those of reCPP-1-PEG nanofibers, *w* ≈ 503 ± 89 nm and *h* ≈ 58 ± 13 nm. The elastic modulus of reCnidoin-PEG nanofibers was *E*_Cnidoin + PEG, air_ = 3.9 ± 3.5 GPa. The removal of PEG by washing with water resulted in a drastic decrease in the fiber thickness by a factor of 7 to *h* ≈ 8 ± 3 nm, which is less pronounced compared to reCPP-1. This finding suggests that reCnidoin proteins were collapsed after the removal of PEG. The dissipation of reCnidoin on the solid substrate was accompanied by a clear increase in the elastic modulus by a factor of 1.5, *E*_Cnidoin, air_ = 5.7 ± 3.4 GPa. In physiological PBS, the thickness of reCnidoin nanofibers was *h* ≈ 6 ± 4 nm, which is similar to reCPP-1 fibers. In fact, we observed a significant decrease in the elastic modulus by three orders of magnitude in physiological PBS, *E*_Cnidoin, PBS_ = 2.8 ± 3.0 MPa. Nevertheless, the higher elastic modulus of reCnidoin in comparison to reCPP-1 can be attributed to the difference in the dissipation of proteins on solid substrates after the removal of PEG. Previous studies reported that the elastic moduli of the nanofibers based on collagen and silk protein in air to be *E*_collagen, air_ = 1 − 8 GPa and *E*_spider silk, air_ = 4.5 GPa, respectively^48,49^. The elastic moduli of reCPP-1 and reCnidoin nanofibers agree well with these data.

The fact that the fibers were not dissolved even after washing with water and possess the elastic modulus in the order of MPa suggests that reCPP-1 and reCnidoin are able to establish stable nanofibers by spontaneously forming intermolecular disulfide bonds between CRD termini (Fig. 1d). In fact, the significant decrease in the elastic moduli of reCPP-1 and reCnidoin nanofibers in physiological PBS seems to agree well with the previous report on collagen fibers in PBS, *E*_collagen, PBS_ = 0.1 − 0.3 MPa^49^. As presented in Supporting Information S15, reCnidoin could not form continuous nanofibers when free CRD peptides were mixed, indicating that the intermolecular crosslinking between reCnidoin proteins was disturbed by the competitive binding of free CRDs. As our data implied that the *Hydra* nematocyst proteins, reCPP-1 and reCnidoin, spontaneously form uniform and stable nanofibers via naturally occurring CRDs not only in air but also in physiological PBS, we examined if these nanofibers can be used for the stable culture of human mesenchymal stem cells on reCPP-1 and reCnidoin nanofibers (Supporting Information S16). After 20 d of incubation, we found that ≈ 95% of cells showed an immunoreactivity to the antibody to the stem cell marker STRO-1 (Supporting Information S17)^50,51^.

## Conclusions

In this study, we propose a new, crosslinker-free nanofiber biomaterial based on the nematocyst capsule proteins of *Hydra*. We expressed recombinant proteins of two recently identified nematocyst capsule proteins, CPP-1 and Cnidoin, in *E. coli*, and prepared nanofibers by electrospinning. As both proteins possess cysteine-rich domains (CRDs), the electrospun fibers are able to spontaneously crosslink via disulfide bonds.

As the viscosity of pure protein solutions was merely *η* ≈ 2 × 10^−3^ Nm^−2^s, we mixed them with 4% (w/v) solution of polyethyleneglycol (Mw = 900 kDa) to fabricate homogeneous and continuous nanofibers. After the optimization of preparative conditions, we electrospun nanofibers. After the removal of PEG by washing with water, we found that protein nanofibers still remained on the surface, confirming the spontaneous crosslinking of CRDs. The remaining fibers possess the elastic moduli of several GPa in air, which agree well with those of collagen and silk protein nanofibers (1–10 GPa). In buffer, the elastic moduli decreased by four orders of magnitude due to hydration.

Since reCPP-1 and reCnidoin can spontaneously form uniform nanofibers that are stable in water without any additional crosslinker, they enable us to use them directly after electrospinning. Finally, we seeded human mesenchymal stem cells. Since about 95% of cells exhibited the immunoreactivity to a stem cell marker (anti STRO-1) after 20 d, reCPP-1 and reCnidoin nanofibers have a potential as new biocompatible materials inspired by the tough and elastic *Hydra* nematocyst structure.

## Supplementary information

Supplementary information
