# Supplementary material for: New Class of Crosslinker-Free Nanofiber Biomaterials from Hydra Nematocyst Proteins
Source: Sci Rep. 2019 Dec 13;9:19116. doi: 10.1038/s41598-019-55655-0 (PMC6910907; doi:10.1038/s41598-019-55655-0)
Supplement: Supplementary file 1 — Supplementary information [file 41598_2019_55655_MOESM1_ESM.docx]

**Supporting Information**

**New Class of Crosslinker-Free Nanofiber Biomaterials from *Hydra* Nematocyst Proteins**

Theresa Bentele^1,#^, Federico Amadei^2,#^, Esther Kimmle^2^, Mariam Veschgini^2^, Philipp Linke^2^, Mariana Sontag^2,‡^, Jutta Tennigkeit^1^, Anthony D. Ho^3,4^, Suat Özbek^1,*^ and Motomu Tanaka^2,4,*^.

^1^ Centre for Organismal Studies, Department of Molecular Evolution and Genomics, Heidelberg University, D69120 Heidelberg, Germany

^2^ Physical Chemistry of Biosystems, Institute of Physical Chemistry, Heidelberg University, D69120 Heidelberg, Germany

^3^ Department of Medicine V, University of Heidelberg, 69120 Heidelberg, Germany

^4^ Center for Integrative Medicine and Physics, Institute for Advanced Study, Kyoto University, 606-8501 Kyoto, Japan

^#^ Equal contribution.

^‡^ Present address: School of Earth and Environmental Sciences, Science Medicine and Health, University of Wollongong, NSW 2522 Wollongong, Australia

^*^ Corresponding authors: suat.oezbek@cos.uni-heidelberg.de, tanaka@uni-heidelberg.de

**CPP1:**

MGTTHPTTKPTKAPTCPKQGKDFYVQIKVLGQGDWSYEMTRVETPGFQDMREKLYDCALQTYADYDFYQDMILLSLDNSTGGFLASFAIRFTKEGDGHLNRLTQAIQAGKFCDVEVAPKFVQCAELDQTMLYPMANCPAPCGGDLNCWPTCDATCCGSQEQTIYIPVAPAPPPPPPPPPPPPPPPPMMSMCAAGCPETCAPSCSPTCCFVQKRWIEKQKAKFQTK QQRYIAPRQKLEHHHHHH

**Cnidoin:**

MAALPGGATFSVPQAVKQLRCPAPCSQSCASSGCSPSCCMNSMPQMPASLSPMMGGCGNQMQGCDQQYMMGGCGGQMQGCGQQMPQMSMGCGGQMQGCGQQMPIMMPGCGAQMQGCGQQMPPLMGGCGGQMQGCGQQMPQMVGGCFGQMVGCGTQTFQSSLKAPCAPNSIGCGQQLRAPMVSMTPGCGGQMQGCGQQMPPMMSGCGGQMQGCGQQSPPMMSGCVGQMQGCGQQLPLMMPGCVGQMQGCGQQMPPMMSGCGGQMQGCGQQMPPMMSGCGGQMQGCGQQIMPMMAPIMPGCGGQMQAGCGGQQEEQMQFKVKLLPPQIYSIQQQQPQQQSQCPPQCQPQTCQIGCPQTCCMQSQPQTAMQMPQPMMV MGGCAPSCQQQCIPSCPRGCCGAFGKKRLEHHHHHH

**S1. Amino acid sequences of recombinant CPP1 and Cnidoin proteins expressed in *E. coli*.**

CRDs, polyhistidine tags, polyproline, and elastic domains were highlighted in yellow, blue, green, and red, respectively.

**
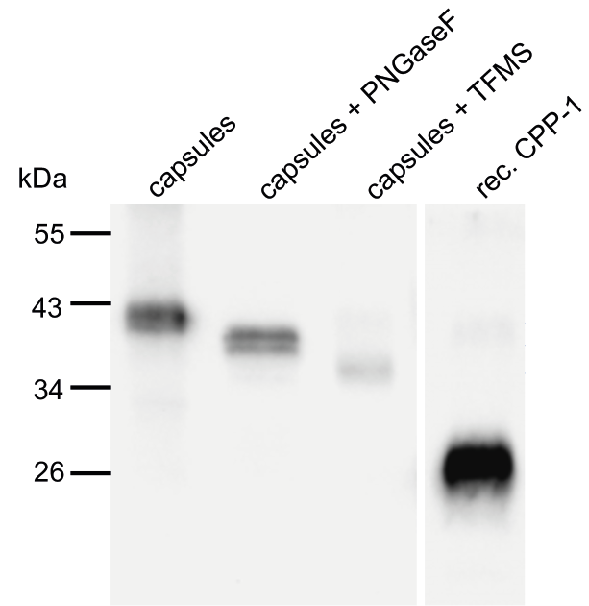
**

**S2. Posttranslational modification of CPP-1 by glycosylation.**

Isolated nematocyst capsules were subjected PNGaseF or trifluoromethanesulfonic acid **(**TFMS) treatment to remove possible N- or O-glycosylation of the mature protein. PNGaseF treatment, which enzymatically removes N-glycosylation reduced the apparent molecular weight of CPP-1 in nematocysts by 4-5 kDa whereas chemical removal of N- and O-glycosylation resulted in an additional shift of the molecular weight by 1-2 kDa. The remaining molecular weight difference to the bacterially expressed CPP-1 protein is suggestive of further modifications like proline hydroxylation.

**
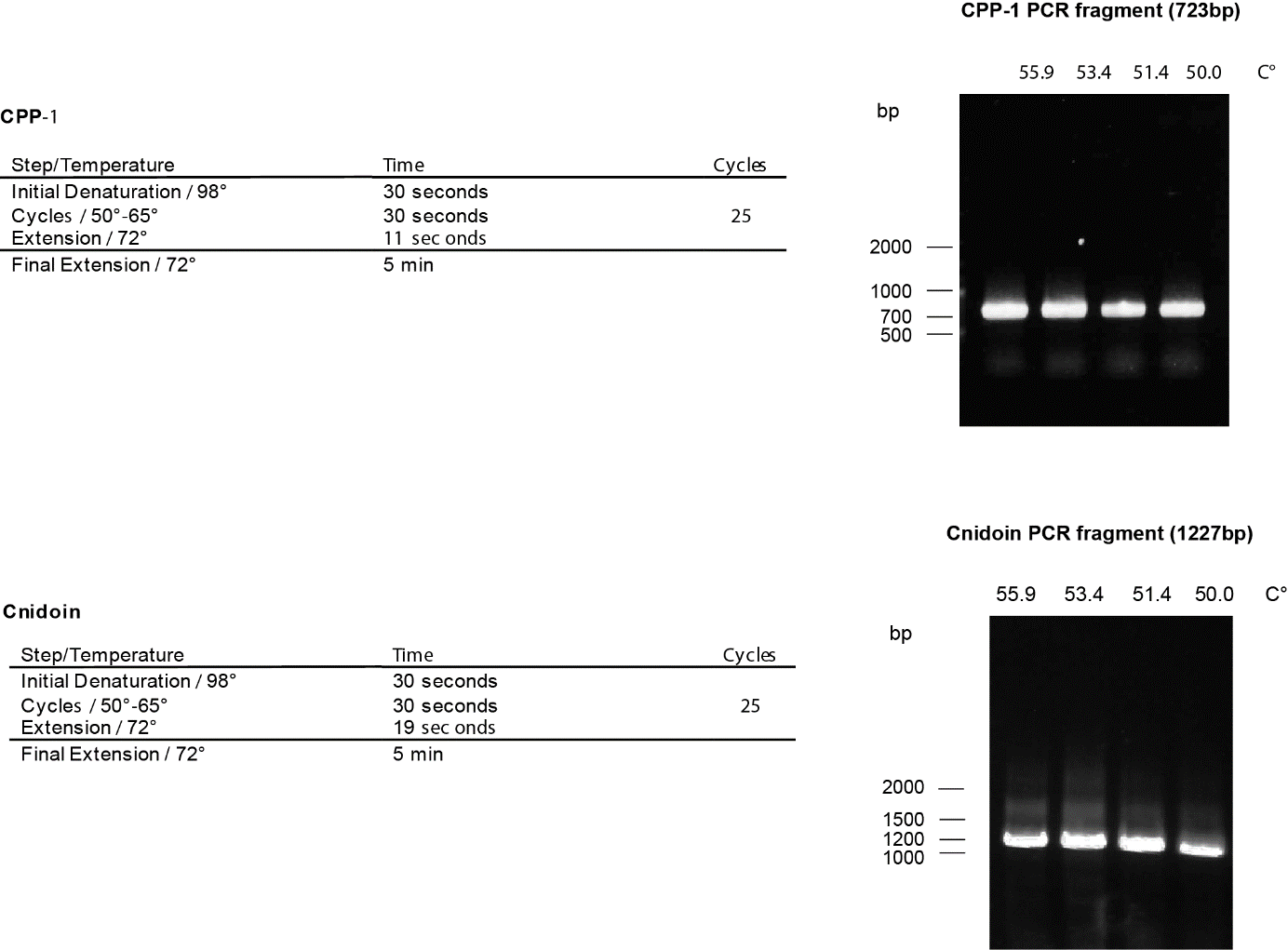
**

**Fig. S3. PCR reaction protocols and gel images of PCR products.**

The recombinant expression of Cnidoin and CPP-1 in *E. coli* BL21 (DE3) was performed from a pET21(+) vector (Invitrogen), which introduces a C-terminal polyhistidine tag. The Cnidoin DNA sequence lacking signal and propeptide sequences was amplified from a full-length cDNA construct described in Beckmann et al. using primers GTT GGA TCC ATG GCT GCA CTT CCG GGA G (forward) and CCC CTC GAG TCT CTT TTT ACC AAA AGC TCC AC (reverse). The PCR reaction was performed using Precisor polymerase (BioCat) with 0.1ng of template and 5pMol/µl of each primer.The CPP-1 DNA sequence lacking signal and propeptide sequences was amplified from a synthetic full-length cDNA inserted into the pcDNA3.1 vector (Biomatik) on the basis of *Hydra* genome and transcriptome data. Primers used for amplification were ATA GAA TTC ATG GGA ACT ACT CAC CCA ACC AC (forward) and GTA CTC GAG TTT CTG TCT CGG AGC AAT ATA TC (reverse). The PCR reaction was performed using Precisor polymerase (BioCat) with 1ng of template and 5pMol/µl of each primer. PCR reaction conditions, gel images of PCR products for each construct are presented.


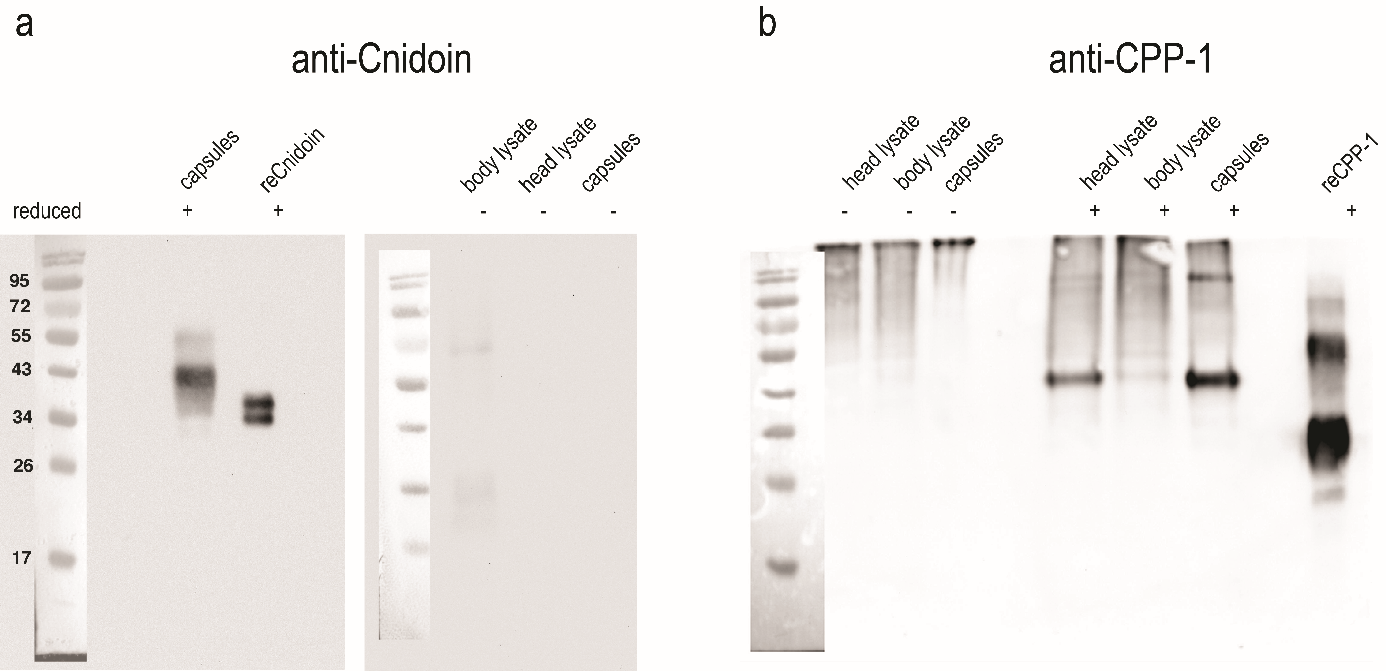


**S4. Uncropped Western blot results.**

(a) Cnidoin and (b) CPP-1 in head lysate, body lysate, and isolated nematocysts. (+) and (−) indicate the presence or absence of β−mercaptoethanol (β−ME) in the sample buffer.


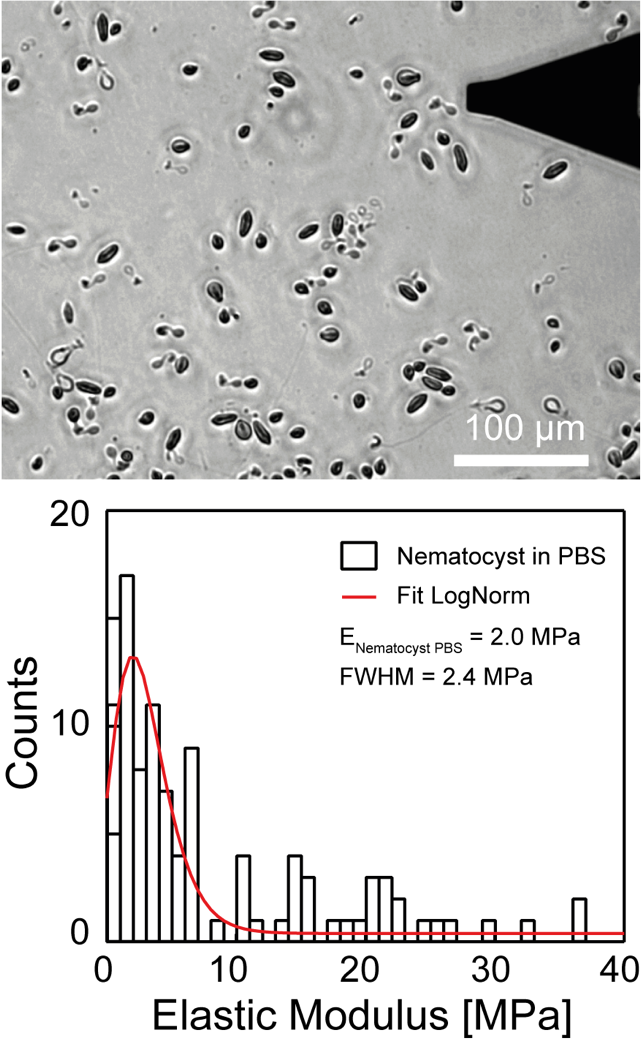


**S5. Effective elastic moduli of *Hydra* nematocysts in PBS.**

Isolated nematocysts were subjected to AFM indentation. The histogram of effective elastic moduli was fitted using a log normal distribution. The peak position and full width at half maximum (FWHM) are presented as the legend.


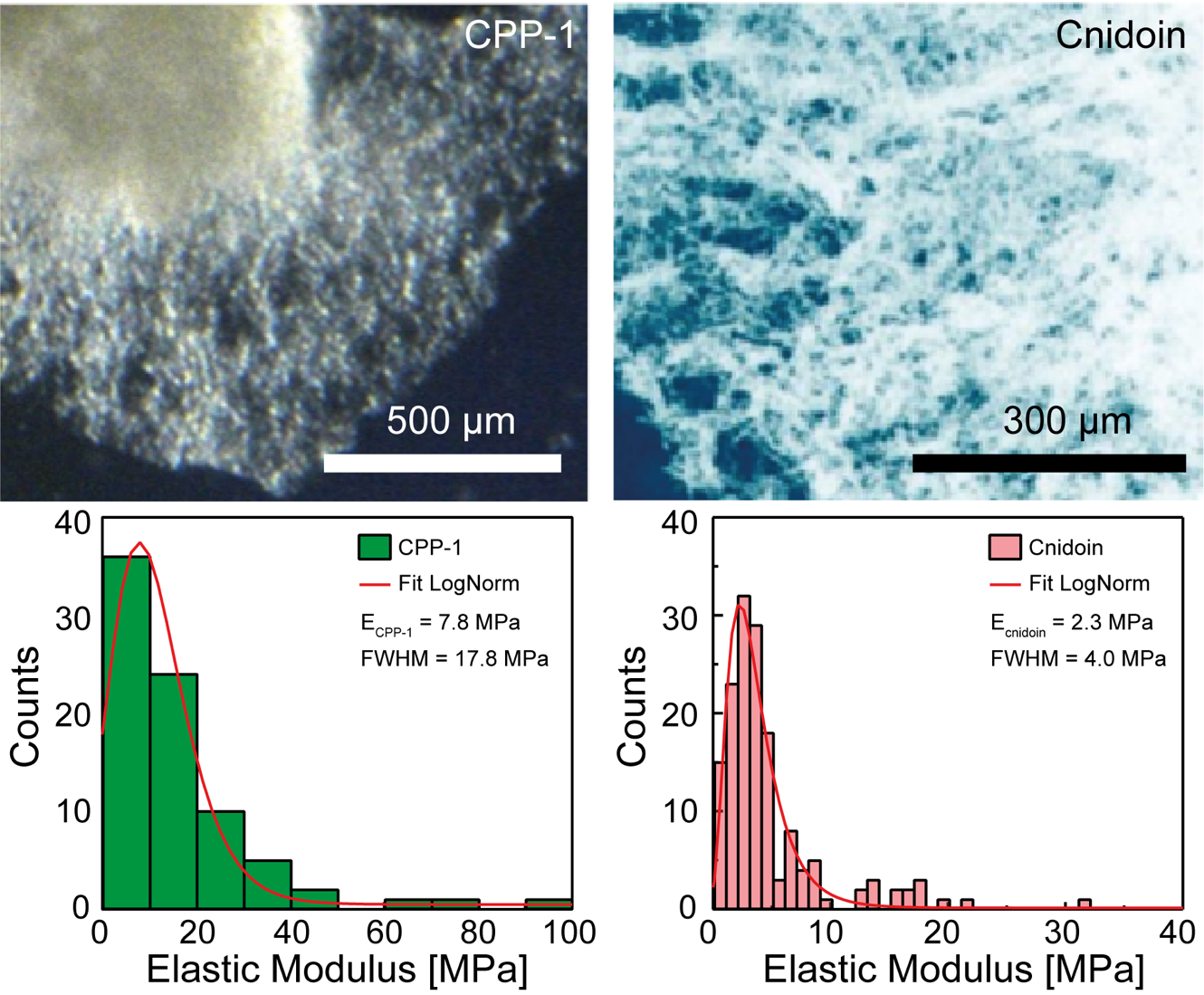


**S6. Effective elastic moduli of recombinant CPP-1 and Cnidoin in PBS.**

Aggregates from purified and oxidized reCPP-1 and reCnidoin proteins were subjected to AFM indentations. The distributions of the effective elastic moduli were fitted using a log normal distribution. The peak positions and FWHM are shown as the legends.


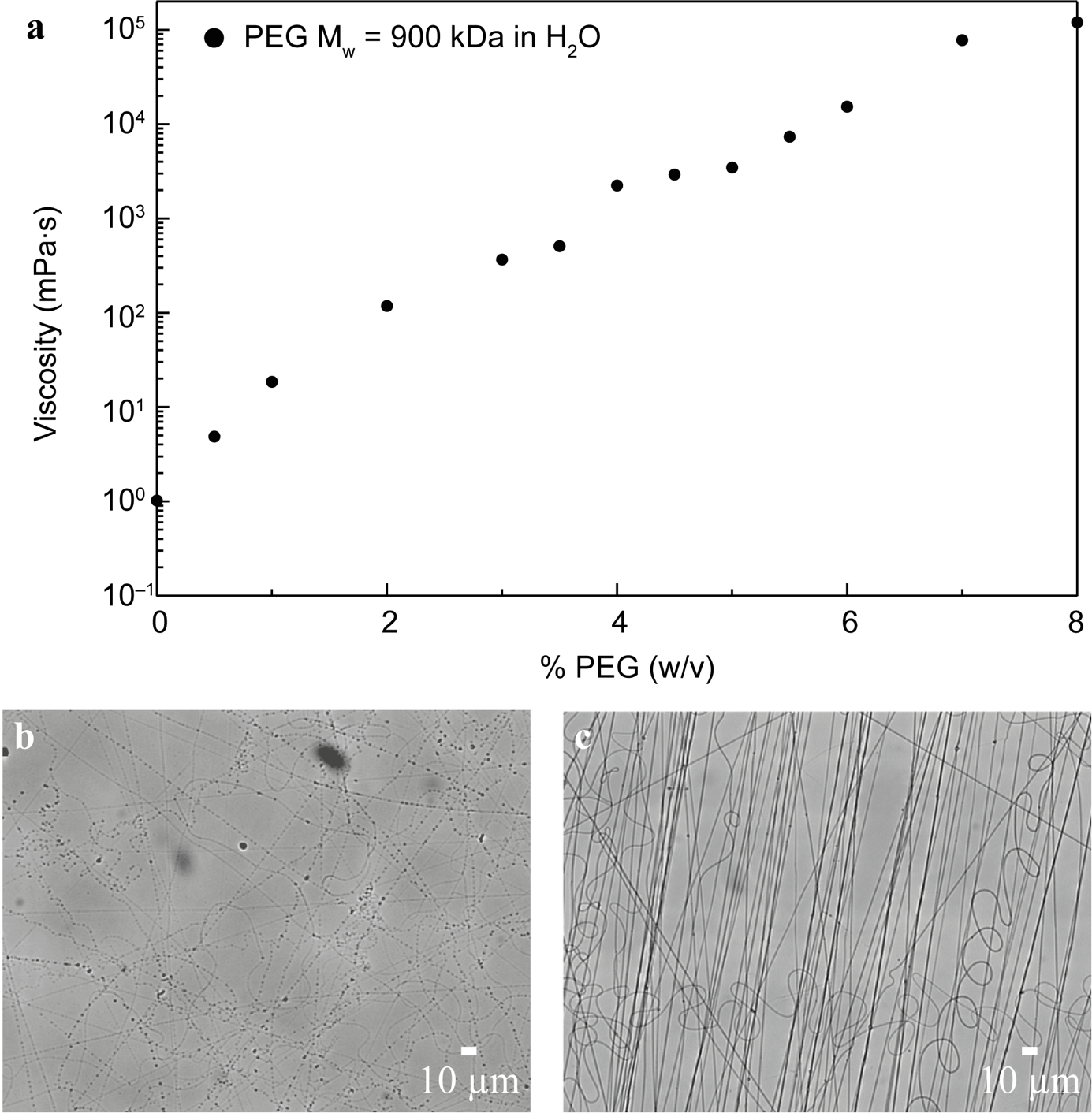


**S7. Influence of viscosity on nanofibers.**

(a) Viscosity of PEG (M_w_ = 900 kDa) as a function of concentration, measured by a self-built falling ball viscometer. Bright field microscopy images of pure PEG fibers with (b) 2 % (w/v) and (c) 4 % (w/v).


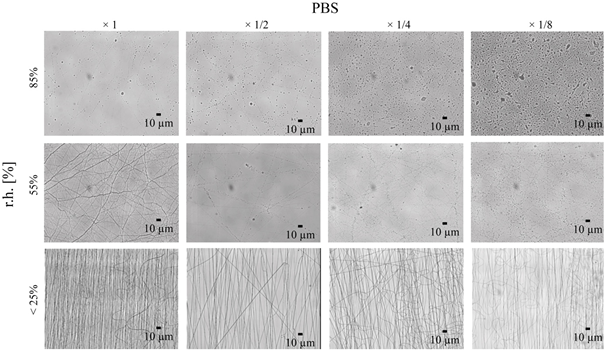


**S8. Influence of ionic strength and humidity on nanofibers.**

Bright field microscopy images of pure PEG fibers (4 % (w/v)) spun at different salt buffer concentrations (× 1, × 1/2, × 1/4, × 1/8) and different air relative humidity conditions (85%, 55%, and < 25%). The relative humidity was controlled by filling the spinning chamber with N_2_.


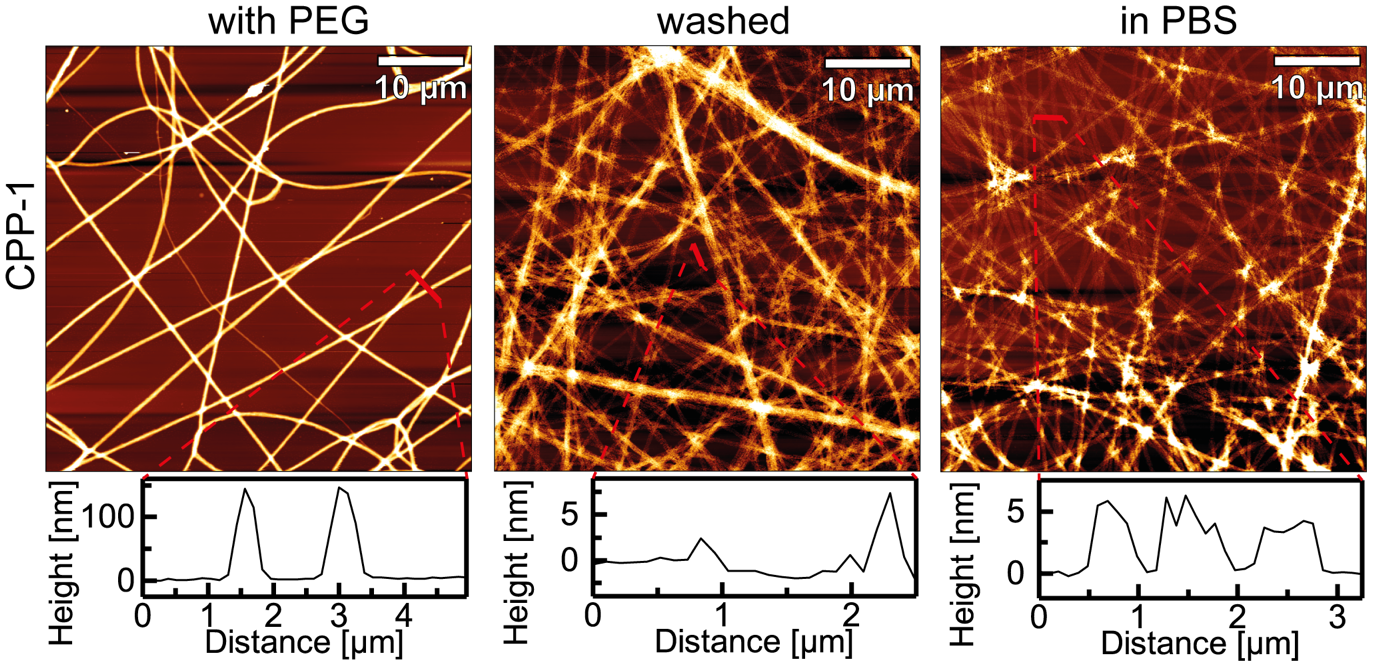


**S9. Height profiles of reCPP-1 nanofibers at different conditions as indicated.**


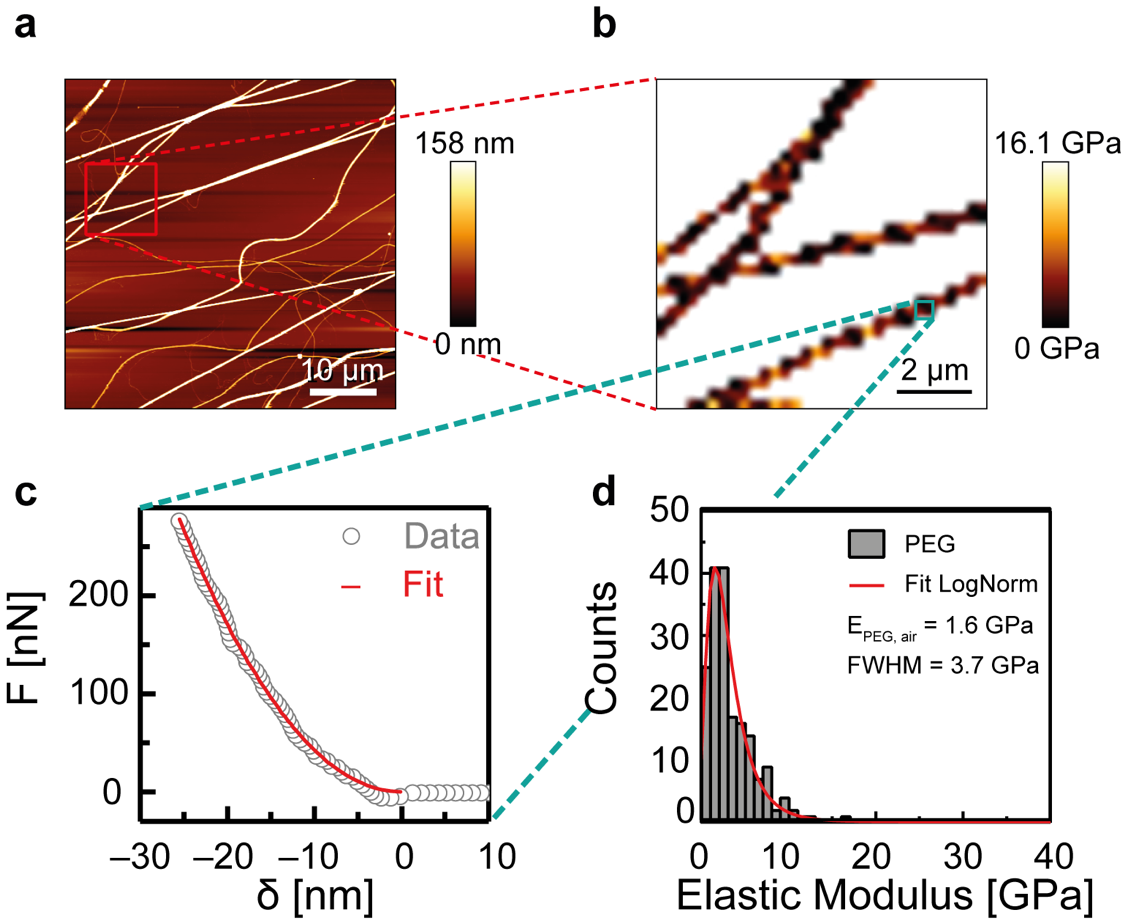


**S10. Elastic modulus of pure PEG fibers in air.**

(a) Surface topography, (b) elasticity map, and (c) a typical force-indentation curve are presented. The log normal fit yields the peak position at 1.6 GPa and FWHM of 3.7 GPa.


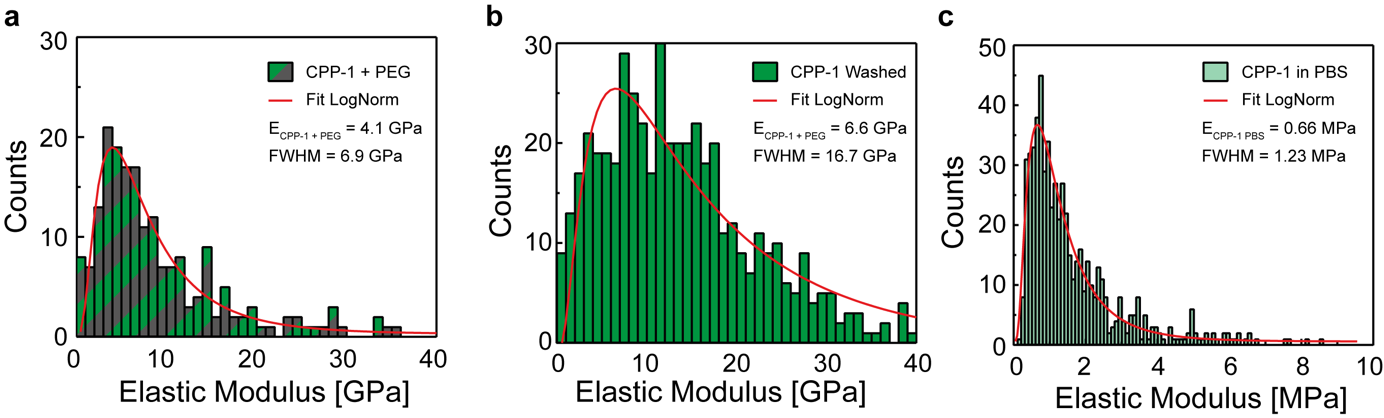


**S11. Statistical distribution of reCPP-1 nanofibers.**

The histograms of each data set to Fig. 4 were fitted with log normal function. The peak positions and full width at half maximum (FWHM) of each dataset are presented.


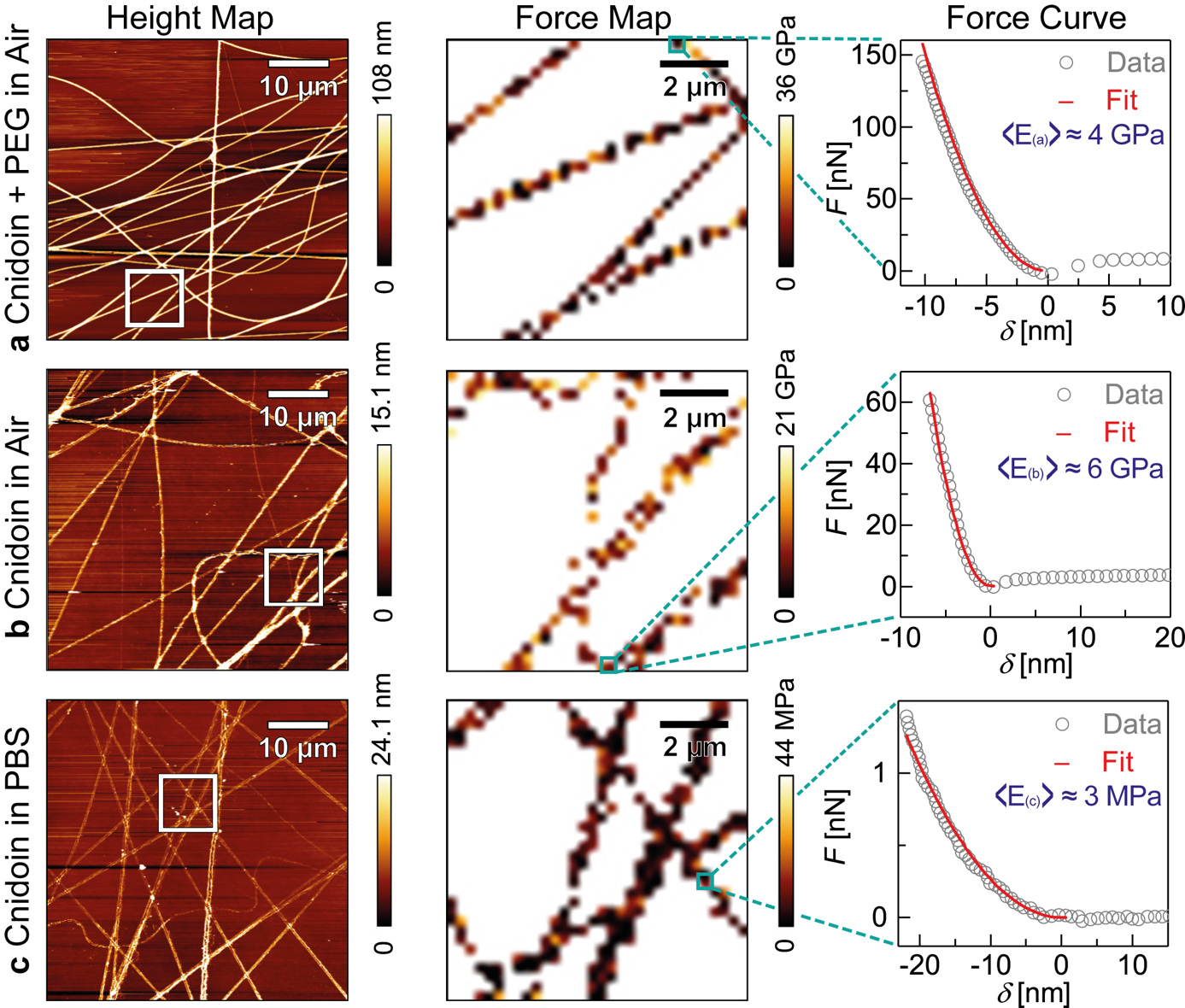


**S12. AFM measurements of electrospun reCnidoin fibers.**

First, a reCnidoin:PEG (1:1) mixture was electrospun and characterized in air (a). Second, the reCnidoin:PEG fibers were washed by water, and the remaining reCnidoin fibers were characterized in air (b), as well as in PBS (c). Each dataset consists of height maps (left column), force maps (middle column), and characteristic force-indentation curves (right column) fitted with the Bilodeau model (red curve).


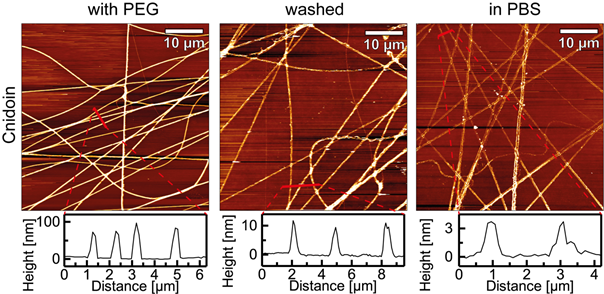


**S13. Height profiles of reCnidoin nanofibers at different conditions as indicated.**


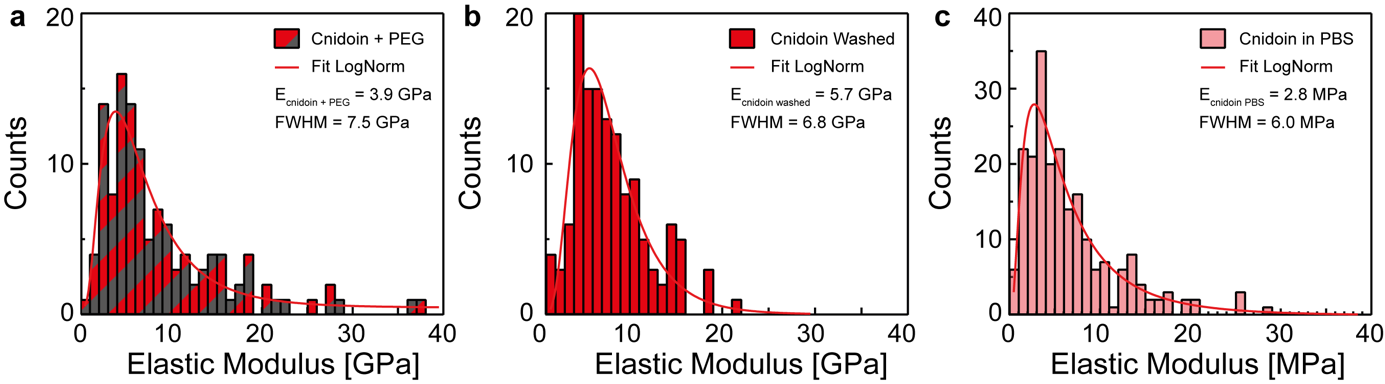


**S14. Statistical distribution of reCnidoin nanofibers.**

The histograms were fitted with log normal function. The peak position and full width at half maximum (FWHM) of each dataset are presented.


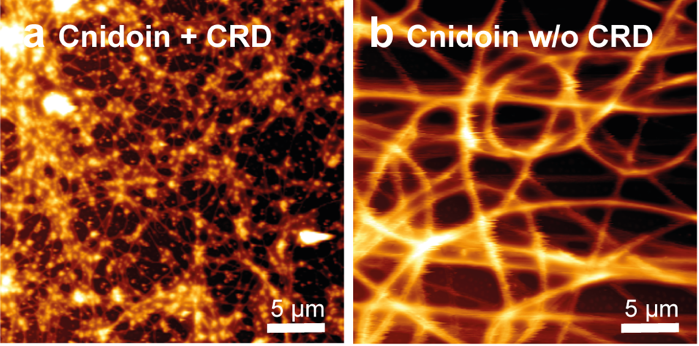


**S15. AFM imaging of electrospun reCnidoin nanofibers with and without CRD peptides.**

(a) reCnidoin and N-CRD peptides were mixed together in a 1:3 molar ratio and electrospun in addition to 4 % PEG (w/v) and (b) Electrospun nanofibers containing reCnidoin and PEG without additional CRD peptides.


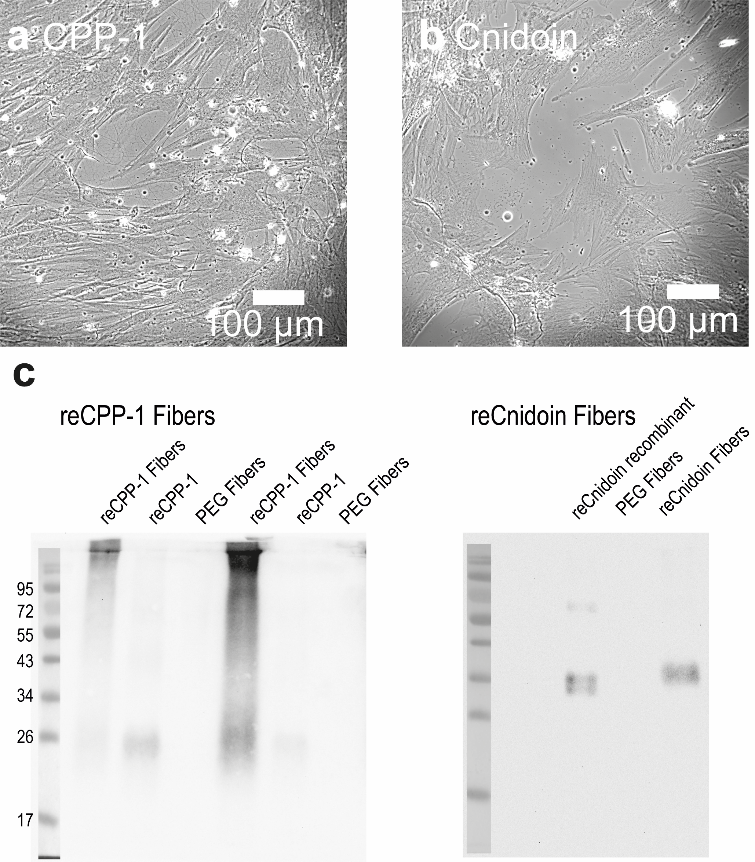


**S16. Human mesenchymal stem cells (hMSC) cultured on protein nanofibers.**

hMSC for 10 d on electro-spun (a) reCPP-1 and (b) reCnidoin fibers. (c) Western Blot of protein fibers after washing and pure PEG fibers (control). Note that the reCPP-1 protein in the fiber sample was partially oligomeric as indicated by additional high molecular weight bands.


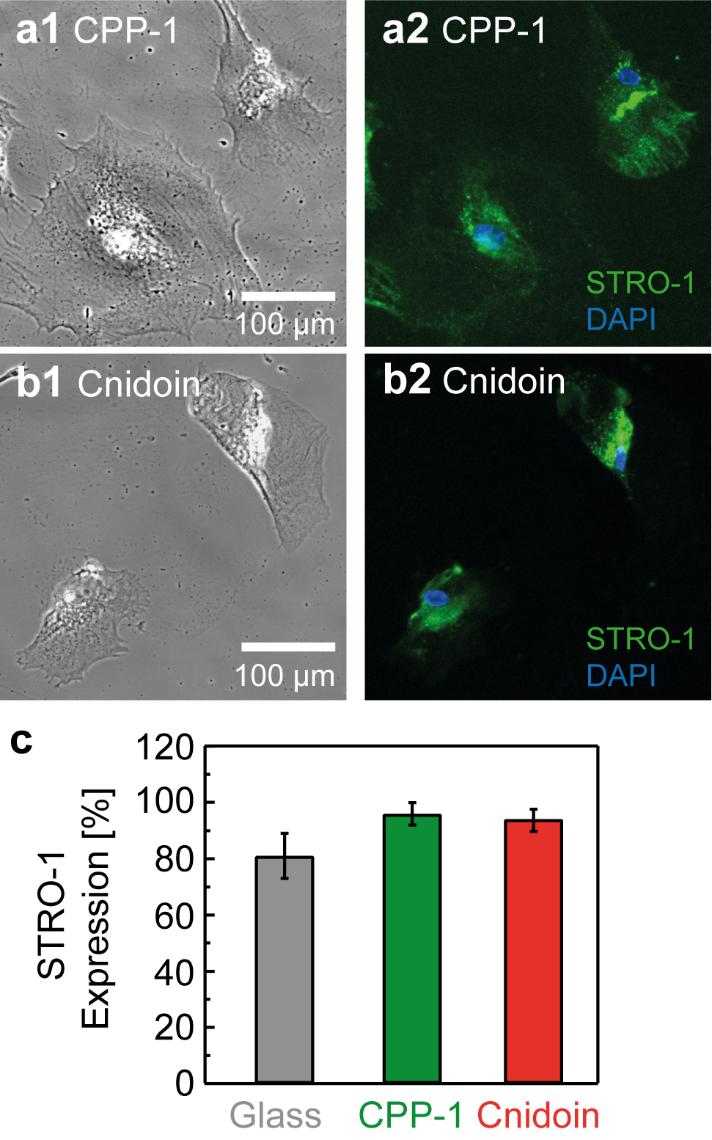


**S17. Maintenance of hMSC on nanofiber substrates.**

Protein nanofibers substrates coated with (a) reCPP-1 and (b) reCnidoin nanofibers for 20 days. Phase-contrast microscopy images (a1 and b1) and the corresponding fluorescence images (a2 and b2) show the expression of STRO-1 (green) in the cytosol of hMSC. Cell nuclei were stained with DAPI (blue). (c) Fractions of hMSC immunoreactive to anti STRO-1, cultured for 20 d on glass (control), reCPP-1 and reCnidoin nanofibers (*N* > 30 for each samples).
